# Biliary Microbiota in Choledocholithiasis and Correlation With Duodenal Microbiota

**DOI:** 10.3389/fcimb.2021.625589

**Published:** 2021-04-29

**Authors:** Jinyan Han, Shuodong Wu, Ying Fan, Yu Tian, Jing Kong

**Affiliations:** Department of General Surgery, Shengjing Hospital of China Medical University, Shenyang, China

**Keywords:** choledocholithiasis, biliary microbiota, duodenal microbiota, duodenal–biliary reflux, antimicrobial resistance

## Abstract

**Background:**

The pathogenesis of choledocholithiasis is closely related to the role of bacteria. However, little is known about the predictive role of bile bacteria in clinical conditions of patients and the compositional and functional characteristics of biliary microbiota in choledocholithiasis.

**Methods:**

To investigate the predictive value of biliary bacteria, clinical data of 488 patients with choledocholithiasis were collected. The predictive value of common bile bacteria to patients’ clinical conditions was analyzed by logistic regression. Samples of bile and corresponding duodenal juice from 10 selected patients with choledocholithiasis were obtained, and the composition and function of microbial communities were analyzed based on *16S* rRNA sequencing and Tax4Fun.

**Results:**

The clinical conditions of patients with choledocholithiasis, such as recurrence, the severity of acute cholangitis, and duration of hospital stay were closely related to different species of bile bacteria as well as antimicrobial-resistant bacteria. Employing *16S* rRNA sequencing, the dominant phyla of biliary and duodenal microbiota were *Proteobacteria* and *Firmicutes*. The top three core microbiota at the genus level were *Escherichia–Shigella, Fusobacterium*, and *Enterococcus*. *Escherichia coli* accounted for the most abundant annotated species in both. Differences in composition between biliary and duodenal microbiota were not significant according to the alpha and beta diversities. Differential abundant features were not found in biliary microbiota indicated by A linear discriminant analysis effective size algorithm. The major pathways identified in biliary and duodenal microbiota were related to membrane transport, translation, replication and repair, carbohydrate and amino acid metabolism. However, no significant difference in those major pathways, as well as antimicrobial-resistance patterns, was observed between biliary and duodenal microbiota.

**Conclusion:**

Our study first demonstrates the predictive contribution of biliary bacteria to the clinical conditions of patients with choledocholithiasis, and then it offers new insights into the compositional and functional features of biliary and duodenal microbiota. Similarities between biliary and duodenal microbiota support the theory of bacterial duodenal–biliary reflux in patients with choledocholithiasis. Meanwhile, when it is impracticable to obtain a bile sample, duodenal juice may be used as an alternative for bacterial culture and susceptibility tests.

## Introduction

Choledocholithiasis has long remained a high-cost and refractory disease with a high rate of recurrence in China. Unlike in the West, choledocholithiasis in China is characterized by stones mainly composed of calcium bilirubinate and forms secondary to bacterial infections and stasis in the biliary tree (Swidsinski and Lee, 2001; Stewart et al., 2002). The classical pathogenesis proposed by Maki has been widely accepted that the hydrolysis of bile components by bacterial enzymes, such as beta-glucuronidase and phospholipase A1, promotes the formation of stones (Maki, 1966; Vítek and Carey, 2012). Thus, it is important to investigate the role of biliary bacteria in this complex disease.

Bile from healthy individuals was considered to be sterile based on traditional microbiological tests (Csendes et al., 1996), whereas many bacteria, including *Escherichia coli, Klebsiella pneumonia, Enterococcus faecium*, and *Enterococcus faecalis*, were identified in the bile or stone samples of patients with choledocholithiasis (Maluenda et al., 1989; Swidsinski and Lee, 2001; Flores et al., 2003). Nevertheless, when it comes to biliary bacteria, attention has mostly focused on the selection of antibiotics for treatment, while other indicative values of specific bacteria to the clinical states are often overlooked. Beyond that, the way of bacterial invasion into the biliary tree has been somehow controversial. Sung et al. described the pathway of the portal–venous system (Sung et al., 1991); however, subsequent studies with both clinical (Liang et al., 2016) and experimental findings (Fukuda et al., 2006) suggested a duodenal–biliary reflux pathway that could facilitate bacterial entry into the bile duct from the duodenum and contribute with the onset of this pathology (Zhang et al., 2015). Unfortunately, this viewpoint has not been widely accepted.

With the advent of next-generation sequencing (NGS), studies on the relationship between gallstone diseases and gut microbiota are gradually emerging (Giordano et al., 2018; Nicoletti et al., 2020). Bacterial identification *via 16S* rRNA not only provides a comprehensive picture of microorganisms in a culture-independent manner but also offers phylogenetic relationships across different taxa from the biliary tree and gut. However, comparative studies on biliary and duodenal microbiota in patients with choledocholithiasis using NGS are limited. Meanwhile, it is noteworthy that fecal microbiota is mostly used as being representative of “gut microbiota” (Wu et al., 2013; Shen et al., 2015; Wang et al., 2017; Wang et al., 2020). The composition of microbes varies along the digestive tract (Donaldson et al., 2016). Recently, it has been highlighted that considering fecal microbiota to be representative of the entire gut microbiota is incorrect. Thus, selective sampling is required to obtain an accurate representation of the gut microbiota (Vasapolli et al., 2019). The biliary tree and duodenum share the same embryological origin and are anatomical connected. Therefore, it is more reasonable to explore the relationship between biliary and duodenal microbiota for understanding their roles in the pathogenesis of choledocholithiasis.

In this study, the first goal is to investigate the predictive value of biliary bacteria to the clinical conditions of patients with choledocholithiasis based on traditional bacterial culture results. The second goal is to compare the structures and function profiles of biliary and duodenal microbial communities in patients with choledocholithiasis using *16S* rRNA sequencing and Tax4Fun.

## Materials and Methods

### Patient Recruitment and Biospecimen Acquisition

For the first goal of this study, clinical data of patients with choledocholithiasis at the Shengjing Hospital of China Medical University were collected from May 2010 to April 2020. The diagnosis was determined by radiographic examinations (ultrasound, CT scan, MRCP, or endoscopic ultrasound). The items collected were demographic data including age, sex, new-onset or recurrence of this disease, clinical symptoms, laboratory tests including the bacterial culture of bile, antimicrobial resistance, white blood cell (WBC), platelet (PLT), C-reactive protein (CRP), total bilirubin (TBil), alanine aminotransferase (ALT), aspartate aminotransferase (AST), alkaline phosphatase (AlkP), gamma-glutamyl transferase (GGT), albumin (ALB), creatine (CR), prothrombin time and international normalized ratio (PT-INR), length of hospital stay, ICU admission, and mortality. Diagnostic criteria and severity grading assessments for acute cholangitis were established based on the Tokyo Guidelines 2018 (TG18) (Kiriyama et al., 2018). Inclusive criteria included: bacterial culture results from bile were available; bile samples were documented in the surgical records as being aseptically derived from the bile duct during bile duct exploration. Patients with missing information or co-morbidities of other biliopancreatic diseases such as biliary or pancreatic carcinoma were excluded.

In total, 488 patients were eligible for this study, 180 of whom had antimicrobial-resistant bacteria cultured in their bile samples (**Supplementary Table S1**). Bacteria other than Escherichia coli, Klebsiella pneumoniae, Enterococcus faecium, and Enterococcus faecalis were relatively rare in the biliary bacterial culture results, so those bacteria were grouped together for analysis as “other bacteria”. A microbiological culture that showed the presence of two or more types of organisms was labeled as “mixed culture”.

For the second goal, bile samples from 10 patients with choledocholithiasis at this hospital were collected for *16S* rRNA sequencing. Information on patients is provided in **Supplementary Table S2**. The diagnosis was also determined by radiographic examinations. Exclusion criteria were as follows: the intake of antibiotics or proton-pump inhibitors within the previous four weeks, co-morbidities with other biliary or gastrointestinal diseases such as a duodenal diverticulum, a history of ERCP or other biliary or gastrointestinal surgeries, signs of Grade II or III acute cholangitis requiring urgent biliary drainage, recurrent choledocholithiasis, and migratory stones from the gallbladder. The duodenal juice (3~5ml) was collected during endoscopic retrograde cholangiopancreatography (ERCP). Afterward, the corresponding bile sample (3~5ml) was collected aseptically by intraoperative aspiration from the common bile duct during selective laparoscopic bile duct exploration. Any sample that became contaminated with blood during aspiration was discarded. All of these samples were snap-frozen in liquid nitrogen immediately after sampling and then stored at -80°C until they were transferred to the sequencing facility.

This study was conducted in compliance with the 1975 Declaration of Helsinki and was approved by the Institutional Review Board of Shengjing Hospital of China Medical University. All participants provided written informed consent.

### *16S* rRNA Sequencing

Microbial DNA was extracted from bile and duodenal juice using a QIAamp DNA Mini Kit (Qiagen, Hilden, Germany). Amplicons of the V3–V4 region of the *16S* rRNA gene were constructed using a 341F/806R primer pair. Sequencing was carried out on a HiSeq platform (Illumina, San Diego, CA, USA), generating 250 bp paired-end reads.

### Bioinformatics and Statistical Analysis

Sequences were first trimmed and merged before being clustered into operational taxonomic units (OTUs) at a 97% sequence similarity level using the UPARSE algorithm (Edgar, 2013). Taxonomy was assigned using a Ribosomal Database Project classifier (Wang et al., 2007) and the SILVA *16S* ribosomal database V.132 (Quast et al., 2013; Yilmaz et al., 2014).

Tax4Fun was employed for the predictions of the functional profile of a microbial community based on 16S rRNA sequence data. The OTUs obtained against the Silva V.123 database were transformed into a taxonomic profile of Kyoto Encyclopedia of Genes and Genomes (KEGG) organisms, and normalization of the predictions was performed according to the *16S* rDNA copy number (Aßhauer et al., 2015). KEGG Mapper was used to analyze the metabolic networks of biliary microbiota, with an emphasis on the enriched pathways in each metabolism (Kanehisa and Sato, 2020).

In this study, 10 genera of bacteria were defined as the “common bacteria” in the bile of patients with choledocholithiasis based on the results of traditional bile culture (Swidsinski and Lee, 2001; Gomi et al., 2018; Wang et al., 2018; Grigor’eva and Romanova, 2020) and 16S rRNA sequencing. These common bacteria were divided into two groups according to gram staining: gram-negative group included *Escherichia–Shigella* spp.*, Acinetobacter* spp.*, Klebsiella* spp.*, Aeromonas* spp.*, Bacteroides* spp.*, Morganella* spp.*, Citrobacter* spp., and *Pseudomonas* spp., while gram-positive group included *Enterococcus* spp. and *Clostridium* spp. Spearman rank correlation coefficients were calculated to investigate whether the relative abundance of common biliary gram-negative bacteria was associated with that of the corresponding duodenal gram-negative bacteria from each patient. The common gram-positive bacteria were analyzed in the same way.

The alpha-diversity indices, including ACE, chao1, Shannon, Simpson, and observed species were calculated with QIIME, a bioinformatic pipeline (Caporaso et al., 2010), and the statistical significance of differences was determined using a Wilcoxon rank-sum test. To explore beta-diversity, principal coordinates analysis (PCoA) on weighted Unifrac distances was used. Analysis of molecular variance (Amova) by weighted Unifrac distances, permutational multivariate analysis of variance (PERMANOVA or Adonis), and analysis of similarities (Anosim) by Bray-Curtis distances were used to test the differences between biliary and duodenal microbiota. Core microbiota, which was defined by MicrobiomeAnalyst, referred to the set of taxa that were detected in a high fraction of the population above a given abundance threshold. Core microbiota was illustrated by core heatmaps using MicrobiomeAnalyst (Dhariwal et al., 2017; Chong et al., 2020). A linear discriminant analysis (LDA) effective size (LEfSe) algorithm was introduced to identify the features most likely to explain differences between biliary and duodenal microbiota by LDA score greater than or equal to 4. Plots were generated with Prism 8 (GraphPad Software, San Diego, CA, USA).

A Kolmogorov–Smirnov or Shapiro–Wilk test was used to test the normality of data. Continuous variables with a normal distribution were presented as mean ± standard deviation (SD), and non-normal variables were reported as median (interquartile range). Categorical variables were presented as percentage. Binary or ordinal logistic regression was used to predict the value of biliary bacteria to clinical features according to the types of dependent variables. A Student’s *t*-test, Welch’s *t*-test, or a non-parametric Wilcoxon rank-sum test was used to test statistical significance depending on the distribution of normality and the homogeneity of variance. Statistical analyses were performed using IBM SPSS Statistics V24 (IBM, Armonk, NY, USA) and differences with a two-sided P-value < 0.05 were considered to be statistically significant.

## Results

### Clinical Findings

In this study, *E. coli, K. pneumoniae, E. faecium, and E. faecalis* were the most common bacteria present in bile from patients with choledocholithiasis. In contrast to patients with negative bacterial cultures, those with *E. coli, K. pneumoniae*, or mixed cultures had 4.16, 5.06, and 4.61 times higher chances of choledocholithiasis recurrence, respectively. Patients with *E. coli* and mixed cultures were associated with increased odds for a definite diagnosis of acute cholangitis of 4.43 and 5.62, respectively. The odds of severe cholangitis were increased by 11.59 for those with mixed cultures. In contrast to *E. coli* infection, increased risks for a prolonged hospital stay (OR = 12.75) and transfer to the intensive care unit (ICU) (OR = 3.99) were observed among patients exposed to other bacteria, such as *A. baumannii* or *P. aeruginosa* infection. Mixed cultures also raised the likelihood of being transferred to the ICU by 17.80. Having an infection of *E. faecalis* in bile increased the odds for death by 29.33 (**Table 1**). In contrast to patients with non-antimicrobial resistant bacteria, those with antimicrobial-resistant bacteria showed increased odds of recurrence of choledocholithiasis and severe cholangitis of 3.75 and 5.37, respectively, but had comparable odds of 1.51 for a diagnosis of acute cholangitis (**Table 2**). 

**Table 1:** Logistic Regression Model for Biliary Bacteria Predicting Clinical Conditions of Patients with Choledocholithiasis.

	Onset				Diagnosis of acute cholangitis					Severity of acute cholangitis					Length of hospital stay				Transfer to ICU				Death			
	variables		*P* value	OR* (95% CI**)	variables			*P* value	OR (95% CI)	variables			*P* value	OR (95% CI)	variables		*P* value	OR (95% CI)	variables		*P* value	OR (95% CI)	variables		*P* value	OR (95% CI)
	New-onset	Recurrence			No	Suspected	Definite			I	II	III			< 30days	≥ 30days			No	Yes			No	Yes		
***E. coli***	101	60	*0.026*	4.16 (1.19~14.53)	59	12	90	*0.002*	4.43 (1.76~11.19)	48	28	14	0.264	3.64 (0.38~34.92)	85	5	Ref		79	11	Ref		88	2	Ref	
***K. pneumoniae***	36	26	*0.015*	5.06 (1.36~18.75)	35	3	24	0.162	2.04 (0.75~5.56)	10	6	8	0.101	7.14 (0.68~74.74)	21	3	0.249	2.43 (0.54~10.98)	18	6	0.126	2.39 (0.78~7.33)	21	3	0.052	6.29 (0.99~40.03)
***E. faecium***	50	5	0.645	0.70 (0.15~3.20)	33	7	15	0.406	1.54 (0.55~4.30)	10	3	2	0.524	2.23 (0.19~26.08)	12	3	0.068	4.25 (0.90~20.10)	12	3	0.417	1.80 (0.44~7.38)	13	2	0.067	6.77 (0.88~52.30)
***E. faecalis***	11	4	0.271	2.55 (0.48~13.46)	7	3	5	0.207	2.33 (0.63~8.65)	4	1	0	–		5	0	–		4	1	0.615	1.80 (0.18~17.56)	3	2	*0.004*	29.33 (3.02~284.72)
**Others**	33	7	0.595	1.49 (0.35~6.39)	22	4	14	0.206	1.99 (0.69~5.75)	8	3	3	0.308	3.57 (0.31~41.14)	8	6	*<0.001*	12.75 (3.17~51.22)	9	5	*0.032*	3.99 (1.13~14.10)	12	2	0.057	7.333 (0.943~57.003)
**Mixed cultures**	79	52	*0.017*	4.61 (1.31~16.23)	40	11	80	*<0.001*	5.62 (2.19~14.40)	21	28	31	*0.034*	11.59 (1.20~112.17)	71	9	0.186	2.16 (0.69~6.72)	23	57	*<0.001*	17.80 (8.04~39.42)	77	3	0.561	1.71 (0.28~10.53)
**Negative**	21	3	Ref***		17	2	5	Ref		4	1	0	Ref		5	0	–		5	0	–		5	0	–	

*OR, odds ratio; **CI, confidence interval; ***Ref, reference value in logistic regression. Underlined certain P-values is to emphasize that they are < 0.05, indicating that they are statistically significant.

**Table 2:** Logistic Regression Model for Biliary Antimicrobial Resistant Bacteria Predicting Clinical Conditions of Patients with Choledocholithiasis.

		Onset				Diagnosis of acute cholangitis					Severity of acute cholangitis					Length of hospital stay				Transfer to ICU				Death			
		variables		*P*	OR* (95% CI**)	variables			*P*	OR (95% CI)	variables			*P*	OR (95% CI)	variables		*P*	OR (95% CI)	variables		*P*	OR (95% CI)	variables		*P*	OR (95% CI)
		New-onset	Recurrence			No	Suspected	Definite			I	II	III			< 30 days	≥ 30 days			No	Yes			No	Yes		
**Antimicrobial resistant bacteria**	Yes	89	91	*<0.001*	3.75 (2.52~5.59)	64	21	95	*0.023*	1.51 (1.06~2.16)	23	29	43	*<0.001*	5.37 (3.18~9.09)	72	23	*<0.001*	14.38 (4.17~49.51)	61	34	0.965	1.01 (0.59~1.75)	86	9	0.075	2.78 (0.90~8.59)
	No	242	66	Ref***		149	21	138	Ref		82	41	15	Ref		135	3	Ref		89	49	Ref		133	5	Ref	

*OR, odds ratio; **CI, confidence interval; ***Ref, reference value in logistic regression. Underlined certain P-values is to emphasize that they are < 0.05, indicating that they are statistically significant.

### Composition of Biliary and Duodenal Microbiota

After sequencing, an average of 46,345 effective sequences from each sample was retrieved and taxonomically annotated, revealing 3,545 different phylotypes (OTUs) belonging to 32 different phyla and 323 genera.

**Figure 1:**
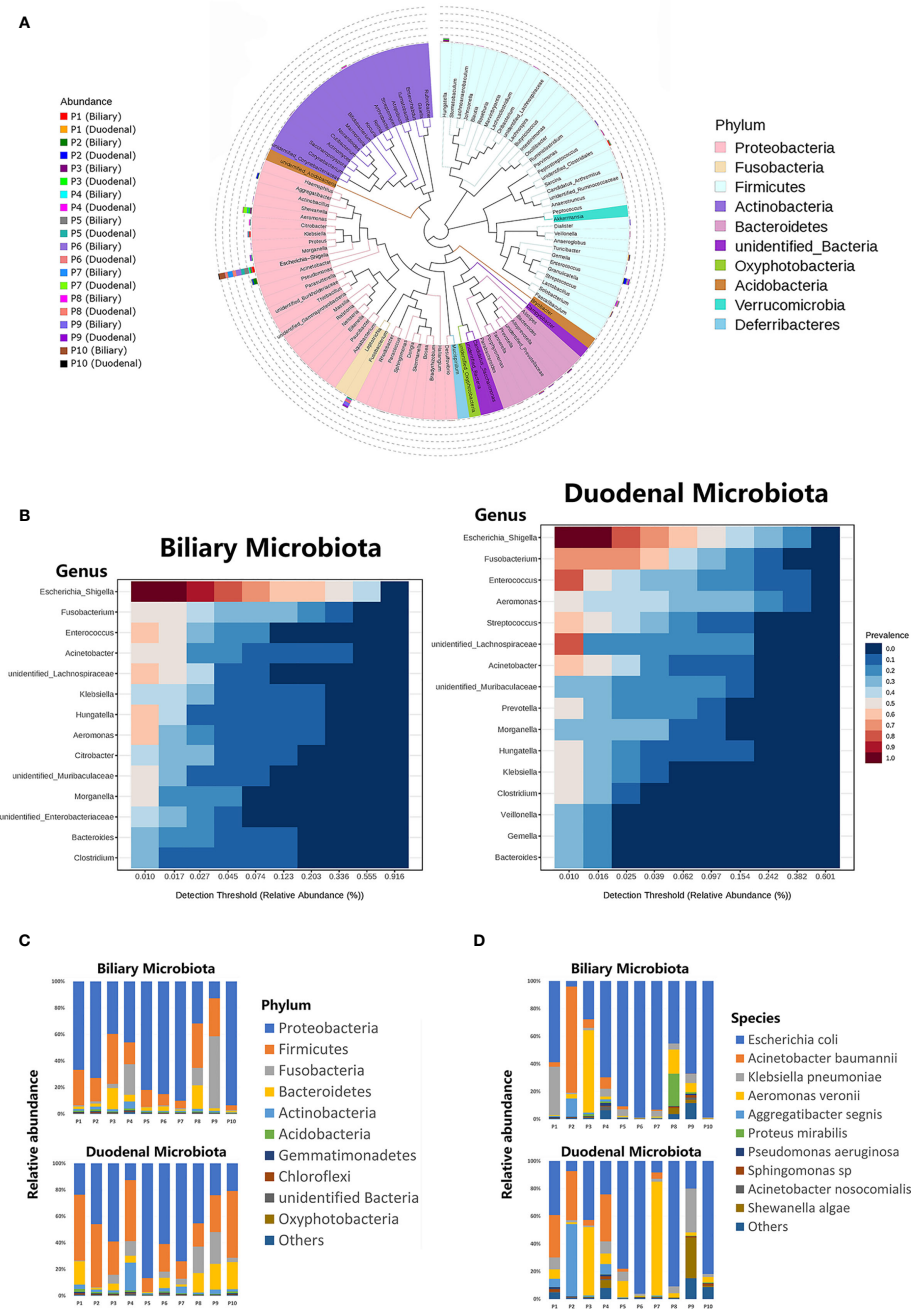
Composition of biliary and duodenal microbiota of patients with choledocholithiasis. **(A)** Phylogenetic trees for biliary and duodenal microbiota (“P” stands for patient). **(B)** Core microbiota of biliary and duodenal microbiota. **(C)** Microbial communities of biliary tree and duodenum at the phylum level. **(D)** Microbial communities of biliary tree and duodenum at the species level in the phylum *Proteobacteria*.

The phylogenetic trees for both the biliary and duodenal microbiota are outlined in a cladogram (**Figure 1A**). The dominant phyla were *Proteobacteria* and *Firmicutes*. The genera *Escherichia–Shigella, Fusobacterium, Aeromonas, Enterococcus*, and *Acinetobacter* were highly abundant in all samples. The core microbiota analyzed in the MicrobiomeAnalyst platform was similar between biliary and duodenal samples at the genus level, with the top three being *Escherichia–Shigella, Fusobacterium*, and *Enterococcus*. However, the duodenal microbiota displayed a more diverse core composition than the biliary microbiota (**Figure 1B**). The major phyla observed in the biliary and duodenal microbiota were both *Proteobacteria* (59.61% and 44.50%, *P* = 0.173) and *Firmicutes* (18.24% and 30.42%, *P* = 0.080) (**Figure 1C**). Of the annotated species in the phylum *Proteobacteria*, *E. coli* accounted for the most abundant in both biliary and duodenal microbial communities (72.60% and 53.88%, *P* = 0.284) (**Figure 1D**). 

### Biliary Microbiota Shared a Compositional Similarity to Duodenal Microbiota

**Figure 2:**
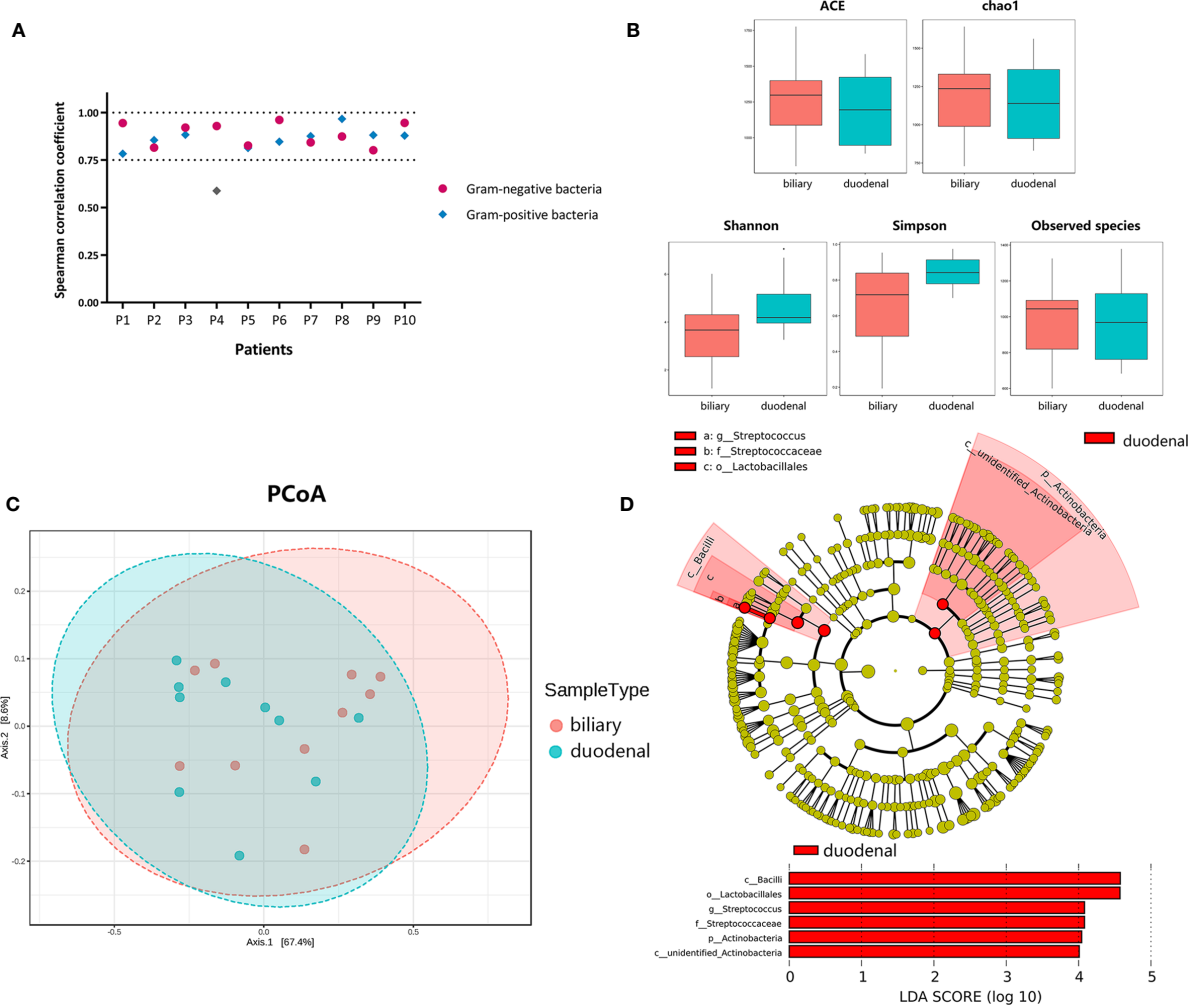
Differences in bacterial communities of biliary and duodenal microbiota of patients with choledocholithiasis. **(A)** Spearman correlation coefficients between biliary and duodenal samples in each patient. **(B)** Differences of microbial alpha-diversity between biliary microbiota and duodenal microbiota. Alpha-diversity was visualized by analysis of Box plots of ACE, chao1, Shannon, Simpson, and observed species index. **(C)** Differences of microbial beta-diversity between biliary and duodenal microbiota in patients. Beta-diversity was visualized by analysis of principal coordinates using weighted Unifrac distances. **(D)** LEfSe cladogram and LDA scores of differential features between biliary and duodenal microbiota.

To investigate correlations between biliary and duodenal microbiota, the correlation coefficients of several Gram-negative and Gram-positive microorganisms, which were commonly isolated from bacterial cultures or the sequencing of bile from patients with choledocholithiasis with/without acute biliary infections, were first compared respectively. Interestingly, Spearman correlation coefficients were positive and statistically significant (Spearman rank correlation, *P* < 0.01) between biliary and duodenal bacteria in all individuals except for a set of Gram-positive bacteria from one patient (**Figure 2A**). Alpha-diversities revealed no significant differences in enrichment and evenness between biliary and duodenal microbiota (ACE: *P* = 0.579; chao1: *P* = 0.796; Shannon: *P* = 0.089; Simpson: *P* = 0.070; observed species: *P* = 0.921, by Wilcoxon rank-sum test) (**Figure 2B**) . Principal coordinates analysis showed no distinct clustering in the biliary and duodenal microbiota, and no significant difference in microbial composition was observed (Amova: *P* = 0.509; Adonis: R^2^ = 0.049, *P* = 0.478; Anosim: R = -0.019, *P* = 0.549) (**Figure 2C**). A LEfSe algorithm indicated the genus *Streptococcus* and an unidentified genus of *Actinobacteria* as the microbial features characterizing duodenal juice from bile in patients with choledocholithiasis; however, no taxa were determined by LEfSe to be characteristic of the biliary microbiota compared with the duodenal microbiota (**Figure 2D**).

### Biliary Microbiota Shared a Functional Similarity to Duodenal Microbiota

**Figure 3:**
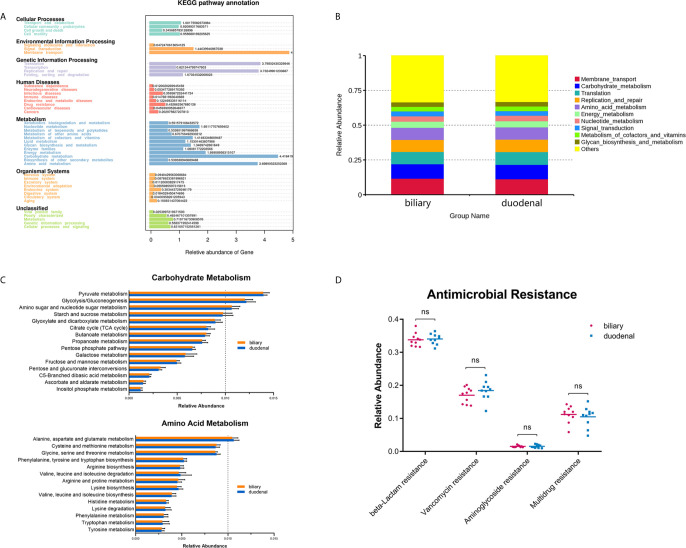
Prediction of functional capacities of biliary and duodenal microbiota of patients with choledocholithiasis. **(A)** KEGG pathway annotation and relative abundance of genes. **(B)** Predicted functional profiles between biliary and duodenal microbiota. **(C)** Relative abundance of the subordinate pathways of carbohydrate metabolism and amino acid metabolism. **(D)** Comparison of gene sets associated with antimicrobial resistance between biliary and duodenal microbiota. ns, not significant.

Functional profiles were obtained through Tax4Fun based on a KEGG database. The major identified pathways of biliary and duodenal microbiota were related to membrane transport, translation, replication and repair, carbohydrate metabolism, and amino acid metabolism (**Figure 3A**). Like microbial composition, differences in major predicted functional profiles between biliary and duodenal microbiota were not significant (**Figure 3B** and **Supplementary Table S3**). Subordinate pathways of carbohydrate metabolism and amino acid metabolism were also analyzed and compared. In both groups of biliary and duodenal microbiota, pyruvate metabolism, glycolysis/gluconeogenesis, amino sugar, and nucleotide sugar metabolism were enriched in carbohydrate metabolism, while alanine, aspartate, and glutamate metabolism were enriched in amino acid metabolism (relative abundance > 1%) (**Figure 3C**). However, no significant distinction was observed between biliary and duodenal microbiota (**Supplementary Tables S4**, **S5**). KEGG Mapper was used to analyze pathways of the aforementioned metabolism of biliary microbiota, with an emphasis on the enriched pathways in each metabolism. Pyruvate oxidation and Embden-Meyerhof pathway were more active in pyruvate metabolism and glycolysis/gluconeogenesis (**Supplementary Figures S1**, **S2**). The pathways for the biosynthesis of UDP-N-acetyl-alpha-D-glucosamine (UDP-GlcNAc) and UDP-glucose were enriched in amino sugar and nucleotide sugar metabolism (**Supplementary Figure S3**). Meanwhile, enriched pathways in alanine, aspartate, and glutamate metabolism were related to the conversion of L-aspartate to fumarate, and L-glutamate to L-glutamine, and its downstream metabolism (**Supplementary Figure S4**). Given that antimicrobial resistance has become a major problem for the treatment of choledocholithiasis and cholangitis, the predicted functions of antimicrobial resistance were compared. However, no significant difference was found in beta-lactamase resistance (Student’s *t*-test, *P* = 0.770), vancomycin resistance (Student’s *t*-test, *P* = 0.256), aminoglycoside resistance (Student’s *t*-test, *P* = 0.883) and multidrug resistance (Student’s *t*-test, *P* = 0.564) between biliary microbiota and duodenal microbiota (**Figure 3D**).

## Discussion

The advent of NGS, together with bioinformatics-based analysis, has revealed a brand-new picture of the human microbiome. Research on gut microbiota has provided novel understandings and perspectives of many diseases. Because the pathogenesis of choledocholithiasis is closely related to bacteria, a deeper investigation of the biliary microbiota is required. Our findings highlighted the indicative values of common bacteria to the clinical conditions of patients with choledocholithiasis based on clinical data. Furthermore, we revealed similarities in the structure and functions of bacterial communities between the biliary tree and duodenum with the help of *16S* rRNA sequencing.

The overall microbial composition at the phylum and genus levels, and even at an identifiable species level, illustrated similarity in abundances with similar alpha and beta diversities between biliary and duodenal microbiota. *Proteobacteria* and *Firmicutes* were the dominant phyla, with *Escherichia–Shigella, Fusobacterium*, and *Enterococcus* the major genera. Even though the interpretation of *16S* sequence data for assigning a definitive bacterial species was limited (Church et al., 2020), *E. coli* was found to be the most abundant species, which was consistent with traditional culture data. LEfSe revealed that no taxon from the biliary microbiota was found to be differentially more abundant as compared to the duodenal microbiota, suggesting that bile shared similar bacterial communities with duodenal juice in patients with choledocholithiasis. A previous study reached a similar conclusion (Ye et al., 2016). However, based on a larger number of samples, more precise OUT annotations according to a continuously updated SILVA rRNA database (Balvočiūtė and Huson, 2017), and multiple means of analysis, this study might provide more comprehensive and convincing information. Our findings for biliary microbial communities were consistent with those of a previous study on the biliary microbiota in patients with new-onset and recurrent choledocholithiasis (Chen et al., 2019). However, it has only recently become evident that bile microbiota in the human gallbladder in individuals without any hepatobiliary disease was composed of the following phyla: *Firmicutes, Bacteroidetes, Actinobacteria*, and *Proteobacteria*, with *Proteobacteria* having a lower proportion than *Bacteroidetes* (Molinero et al., 2019). In comparison, the altered distribution of *Proteobacteria* and *Bacteroidetes* appeared to be a major difference in the biliary microbiota of patients with choledocholithiasis and those without any hepatobiliary disease.

What caused the compositional changes? Compared with recent studies on duodenal microbiota in healthy populations (Vasapolli et al., 2019; Leite et al., 2020), the dominant phyla, which were *Proteobacteria* and *Firmicutes*, did not differ from those in patients with choledocholithiasis in this study. Nevertheless, the microbiota of the bile duct shifted toward compositional and functional parallels with the duodenum. Endoscopic sphincterotomy (ES) is believed to be a major risk factor for the recurrence of choledocholithiasis (Tranter and Thompson, 2002; Dumonceau et al., 2020). This “mechanical” enlargement of the orifice of the common bile duct (CBD) allows bacteria to enter into the bile duct from the duodenum, a condition known as duodenal–biliary reflux. However, Shen et al. discovered no substantial difference in the microbial distribution of bile from patients with and without a history of ES in patients with choledocholithiasis (Shen et al., 2015). This implied the presence of an altered microbial microecology in the biliary tree in patients with choledocholithiasis prior to ES, most likely due to a “pathological” impairment of the barrier function of the sphincter of Oddi. Fukuda et al. revealed that the enlarged orifice of the CBD due to the loss of major duodenal papilla allowed bacterial reflux from the duodenum into the CBD, promoting the development of choledocholithiasis (Fukuda et al., 2006). Therefore, coupled with what has been found in our study, we suggested that the alterations in the biliary microecology in patients with choledocholithiasis might be caused by bacterial duodenal–biliary reflux due to an impairment of the barrier function of the sphincter of Oddi. It might be reasonable that this pathway of bacterial invasion, which circumvents the hepatic immune defense, might ultimately contribute to the formation of common bile duct stones. Of course, this hypothesis has not been confirmed, and further validation is necessary. Nevertheless, care should be taken to maintain the function of the sphincter of Oddi (Chen et al., 2011; Testoni et al., 2016).

Bacteria exhibit different functions in response to changes in their environment. A new colonizing microbiota was expected to respond in a variety of ways to survive and resist the deleterious actions of bile in the biliary tree (Begley et al., 2005; de Jesus et al., 2005; Delmas et al., 2019). Wetter reported on differences in outer membrane characteristics between gallstone-associated bacteria and normal bacterial flora (Wetter et al., 1994). Metabolic functions of biliary microbiota were analyzed in this study because they were seldom explained and could be associated with the pathogenesis of stone formation (Ye et al., 2016). The predicted KEGG pathways significantly enriched in the metabolism of biliary microbiota were carbohydrate metabolism and amino acid metabolism. Further, it was inferred that the enriched pathways in carbohydrate metabolism were associated with a central role for pyruvate. Carbohydrates such as glucose are the most widely used carbon sources for microbes; however, due to a lack of glucose in bile (Guzelian and Boyer, 1974), other components, such as amino acids, are presumably utilized by bacteria for nutrients and energy. The high abundance of pyruvate metabolism might represent the synergistic effects of diverse nutrient utilization. Meanwhile, the predicted active biosynthesis of UDP-GlcNAc of UDP sugar could suggest active metabolic reactions associated with it. For example, the biosynthesis of peptidoglycan, O-antigen, and lipopolysaccharide were all associated with UDP-GlcNAc. Peptidoglycan was found to be involved in the adaptive response which permitted the survival of bacteria in the presence of bile (Hernández et al., 2015). O-antigen was associated with the formation of biofilm on the surface of gallstones (Prouty et al., 2002), while lipopolysaccharide played important roles in the pathogenesis of biliary stone (Li et al., 2014; Yao et al., 2018; Wu et al., 2021). As for alanine, aspartate, and glutamate metabolism, L-aspartate and L-alanine seemed to be used primarily as nutrients and energy, while glutamate, in addition to nutrient supply, might be converted into L-glutamine and utilized in downstream metabolisms, such as nucleotide metabolism. the preferred metabolic pathways could reflected the regulatory decisions of the biliary microbiota for the uptake and utilization of different amino acids in the harsh environment of the bile.

Given the development of an increasing amount of antimicrobial resistance (Buckman and Mazuski, 2019), sample cultures and susceptibility tests should be performed as soon as possible for patients with choledocholithiasis, especially those with acute cholangitis. In addition to the decision for antimicrobial usage, this study demonstrated the predictive value of specific microorganisms from bile for patient conditions. Thus, early intervention measures should be taken when specific bacteria were detected. With a higher positive rate than that of blood, bile was recommended in TG18 as the optimal specimen for culture; it has been suggested that the common duct bile be sent for bacterial culture in all cases of suspected cholangitis in TG18 (Gomi et al., 2018). However, it was noteworthy that TG18 also suggested that most patients with mild acute cholangitis did not require biliary drainage unless they did not respond to initial treatment (Miura et al., 2018). This inconsistency raised the issue of how to obtain bile samples from non-severe cases. Interestingly, our findings highlighted an alternative option for the source of the specimen since the duodenal microbiota shared similarity with biliary microbiota in composition, functions, and even antimicrobial resistance in patients with choledocholithiasis. Furthermore, methods of obtaining duodenal fluid *via* gastroduodenoscopy or gastric tube are relatively less invasive and less risky than those of biliary drainage. Nevertheless, this idea needs to be confirmed by further clinical trials with a larger sample size.

To the best of our knowledge, this is the first study to introduce the predictive roles of biliary bacteria in the clinical status of patients with choledocholithiasis. Meanwhile, it is one of the few studies to introduce the metabolic characteristics of biliary microbiota in patients with choledocholithiasis by the application of *16S* rRNA sequencing, and to support the hypothesis of bacterial duodenal–biliary reflux in choledocholithiasis based on the structure and predicted functions of biliary and duodenal microbiota. An important limitation of our study is the small sample size of the *16S* rRNA amplicon dataset. Therefore, more rationally designed studies with a larger sample size were required to verify our findings. Another limitation is the lack of samples from a healthy group as control. While due to obvious ethical reasons, it is difficult to obtain bile or duodenal samples of the bile duct from healthy individuals. Although sequence data of bile and duodenal juice from healthy individuals are available from the National Centre for Biotechnology Information database (Molinero et al., 2019; Leite et al., 2020), it is more reasonable to obtain samples from Chinese patients, taking into account the factors such as diet and race.

## Conclusions

To sum up, the present study demonstrates the predictive value of biliary bacteria to the clinical conditions of patients with choledocholithiasis and provides new insights into the compositional and functional characteristics of biliary and duodenal microbiota in choledocholithiasis. The similarities between biliary and duodenal microbiota support the theory of bacterial duodenal–biliary reflux as a potential mechanism for the onset of this pathology. Meanwhile, duodenal juice might be a good substitute for bile in bacterial culture and susceptibility tests when it is impracticable to obtain a bile sample. The function of biliary microbiota is a vast treasure trove to be explored. The findings of this study represent an important framework for future investigations into the biliary microbiota as it relates to disease. What has been inferred should be further investigated based on more functional experimental studies with a larger sample size.

## Data Availability Statement

All raw data were deposited in the SRA of the NCBI (https://www.ncbi.nlm.nih.gov/sra) under accession numbers SRR12846125 to SRR 12846144.

## Ethics Statement

The studies involving human participants were reviewed and approved by Institutional Review Board of Shengjing Hospital of China Medical University. The patients/participants provided their written informed consent to participate in this study.

## Author Contributions

JH: Study design, analyzed the data, and wrote the manuscript. SW: Study design and revised the manuscript. YF: Analyzed the data and revised the manuscript. YT: Analyzed the data and prepared the figures. JK: prepared the figures and tables. All authors contributed to the article and approved the submitted version.

## Conflict of Interest

The authors declare that the research was conducted in the absence of any commercial or financial relationships that could be construed as a potential conflict of interest.
