# Supplementary material for: Biliary Microbiota in Choledocholithiasis and Correlation With Duodenal Microbiota
Source: Front Cell Infect Microbiol. 2021 Apr 29;11:625589. doi: 10.3389/fcimb.2021.625589 (PMC8116743; doi:10.3389/fcimb.2021.625589)
Supplement: Supplementary file 1 [file DataSheet_1.docx]

Supplementary Material

# Supplementary Tables

**Supplementary** **Table S1.** Clinical characteristics of patients with choledocholithiasis

| **Parameter** | **Data** |
| --- | --- |
| **Sex** |  |
| male | 246 (50.4%) |
| female | 242 (49.6%) |
| **Age (years)** | 65.5±12.1 |
| **Onset** |  |
| new onset | 358 (73.4%) |
| recurrence | 130 (26.6%) |
| **Acute cholangitis** | 275 |
| suspected | 42 (15.3%) |
| definitive | 233 (84.7%) |
| **Severity of acute cholangitis** | 233 |
| Grade I | 119 (51.1%) |
| Grade II | 67 (28.8%) |
| Grade III | 47 (20.1%) |
| **Laboratory tests** |  |
| WBC (×109/L) | 9.3 (7.6) |
| PLT (×109/L) | 177 (93) |
| CRP (mg/L) | 78.8 (130.17) |
| TBil (μmol/L) | 42.2 (69.5) |
| ALT (U/L) | 93 (177) |
| AST (U/L) | 94 (148.36) |
| AlkP (U/L) | 153 (144.9) |
| GGT (U/L) | 265 (450) |
| ALB (U/L) | 38.8 (7.9) |
| CR (μmol/L) | 68.7 (25.65) |
| PT-INR | 1.1 (0.2) |
| **Bile culture** |  |
| Positive | 464 |
| *Escherichia coli* | 161 (34.7%) |
| *Klebsiella pneumoniae* | 62 (13.4%) |
| *Enterococcus faecium* | 55 (11.9%) |
| *Enterococcus faecalis* | 15 (3.2%) |
| Others (*Acinetobacter baumannii, Pseudomonas aeruginosa, Enterococcus casseliflavus, Klebsiella oxytoca, Enterococcus gallinarum, Enterobacter cloacae, Citrobacter freundii, Citrobacter braakii, Pseudomonas fluorescens, Morganella morganii, Aeromonas hydrophila, Enterococcus avium, Stenotrophomonas maltophilia, Shewanella algae*) | 40 (8.6%) |
| Mixed bacteria | 131 (28.2%) |
| Negative | 24 |
| **Antimicrobial resistant bacteria** | 180 |
| ESBL | 130 (72.2%) |
| HLAR | 26 (14.4%) |
| CRE | 12 (6.7%) |
| VRE | 5 (2.8%) |
| CRABA | 5 (2.8%) |
| CRPA | 2 (1.1%) |
| **Length of hospital stay (days)** | 16 (11) |
| **Transfer to ICU** | 43 (8.8%) |
| **Death** | 14 (2.9%) |

Abbreviations: WBC, white blood cell; PLT, platelet; CRP, C-reactive protein; TBil, total bilirubin; ALT, Alanine aminotransferase; AST, Aspartate aminotransferase; AlkP, Alkaline phosphatase; GGT, Gamma-glutamyl transferase; ALB, albumin; CR, creatine; ESBL, Extended-spectrum β-Lactamase; HLAR, high level aminoglycoside resistant; CRE, Carbapenem-resistant Enterobacteriaceae; VRE, Vancomycin-Resistant Enterococcus; CRABA, carbapenem-resistant Acinetobacter baumannii; CRPA, carbapenem-resistant Pseudomonas aeruginosa.

**Supplementary** **Table S2.** Clinical characteristics of patients whose biospecimens were analyzed by 16S rRNA sequencing

| **Parameter** | **Data** |
| --- | --- |
| **Sex** |  |
| male | 3 |
| female | 7 |
| **Age (years)** | 59.3±7.8 (47 ~ 74) |
| **Laboratory tests** |  |
| WBC (×10^9^/L) | 7.39 (4.53) |
| PLT (×10^9^/L) | 230.5 (125.75) |
| TBil (μmol/L) | 20.35 (49.38) |
| ALT (U/L) | 86 (165.5) |
| AST (U/L) | 66 (104.5) |
| AlkP (U/L) | 154.5 (464) |
| GGT (U/L) | 366 (684.75) |
| ALB (U/L) | 42.05 (4.85) |
| CR (μmol/L) | 55.85 (15.2) |
| PT-INR | 1 (0.1) |
| **Length of hospital stay (days)** | 14 (10) |
| **Transfer to ICU** | None |
| **Death** | None |

**Supplementary** **Table S3.** Predicted functional profiles between biliary microbiota and duodenal microbiota

| **Taxa** | **Relative Abundance** | | ***P* value** |
| --- | --- | --- | --- |
|  | **bile** | **duodenal** |  |
| Membrane transport | 0.116±0.012 | 0.112±0.012 | 0.44 |
| Carbohydrate metabolism | 0.104±0.003 | 0.104±0.004 | 0.98 |
| Translation | 0.088±0.009 | 0.091±0.007 | 0.40 |
| Replication and repair | 0.087±0.008 | 0.090±0.007 | 0.37 |
| Amino acid metabolism | 0.087±0.003 | 0.088±0.004 | 0.55 |
| Energy metabolism | 0.044±0.001 | 0.044±0.001 | 0.71 |
| Nucleotide metabolism | 0.038±0.004 | 0.040±0.003 | 0.41 |
| Signal transduction | 0.035±0.005 | 0.033±0.005 | 0.26 |
| Metabolism of cofactors and vitamins | 0.033±0.001 | 0.034±0.001 | 0.14 |
| Glycan biosynthesis and metabolism | 0.032±0.001 | 0.032±0.004 | 0.86 |

**Supplementary** **Table S4.** Relative abundance of carbohydrate metabolism

| **Taxa** | **Relative Abundance** | | ***P* value** |
| --- | --- | --- | --- |
|  | **bile** | **duodenal** |  |
| Pyruvate metabolism | 0.014±0.0006 | 0.014±0.0004 | 0.89 |
| Glycolysis/Gluconeogenesis | 0.012±0.0008 | 0.012±0.0009 | 0.70 |
| Amino sugar and nucleotide sugar metabolism | 0.011±0.0008 | 0.011±0.0007 | 0.96 |
| Starch and sucrose metabolism | 0.010±0.0009 | 0.010±0.0011 | 0.92 |
| Glyoxylate and dicarboxylate metabolism | 0.009±0.0006 | 0.009±0.0007 | 0.79 |
| Citrate cycle (TCA cycle) | 0.008±0.0004 | 0.008±0.0007 | 0.47 |
| Butanoate metabolism | 0.008±0.0004 | 0.008±0.0005 | 0.45 |
| Propanoate metabolism | 0.008±0.0004 | 0.008±0.0006 | 0.98 |
| Pentose phosphate pathway | 0.007±0.0002 | 0.007±0.0003 | 0.81 |
| Galactose metabolism | 0.006±0.0012 | 0.006±0.0009 | 0.85 |
| Fructose and mannose metabolism | 0.005±0.0003 | 0.005±0.0004 | 0.77 |
| Pentose and glucuronate interconversions | 0.003±0.0005 | 0.003±0.0003 | 0.28 |
| C5-Branched dibasic acid metabolism | 0.002±0.0002 | 0.002±0.0001 | 0.89 |
| Ascorbate and aldarate metabolism | 0.002±0.0002 | 0.001±0.0002 | 0.68 |
| Inositol phosphate metabolism | 0.001±0.0002 | 0.001±0.0002 | 0.73 |

**Supplementary** **Table S5.** Relative abundance of amino acid metabolism

| **Taxa** | **Relative Abundance** | | ***P* value** |
| --- | --- | --- | --- |
|  | **bile** | **duodenal** |  |
| Alanine, aspartate and glutamate metabolism | 0.010±0.0006 | 0.011±0.0006 | 0.53 |
| Cysteine and methionine metabolism | 0.009±0.0004 | 0.009±0.0004 | 0.61 |
| Glycine, serine and threonine metabolism | 0.009±0.0002 | 0.009±0.0004 | 0.35 |
| Phenylalanine, tyrosine and tryptophan biosynthesis | 0.005±0.0004 | 0.005±0.0003 | 0.62 |
| Arginine biosynthesis | 0.005±0.0004 | 0.005±0.0004 | 0.85 |
| Valine, leucine and isoleucine degradation | 0.005±0.0008 | 0.005±0.0012 | 0.68 |
| Arginine and proline metabolism | 0.005±0.0007 | 0.005±0.0005 | 0.70 |
| Lysine biosynthesis | 0.004±0.0005 | 0.005±0.0005 | 0.40 |
| Valine, leucine and isoleucine biosynthesis | 0.004±0.0004 | 0.004±0.0005 | 0.91 |
| Histidine metabolism | 0.003±0.0002 | 0.003±0.0002 | 0.65 |
| Lysine degradation | 0.003±0.0005 | 0.003±0.0006 | 0.88 |
| Phenylalanine metabolism | 0.003±0.0004 | 0.003±0.0005 | 0.94 |
| Tryptophan metabolism | 0.003±0.0007 | 0.003±0.0007 | 0.88 |
| Tyrosine metabolism | 0.003±0.0003 | 0.003±0.0003 | 0.83 |

# Supplementary Figures

**Supplementary** **Figure S1**. Enriched pathways in pyruvate metabolism of biliary microbiota in patients with choledocholithiasis


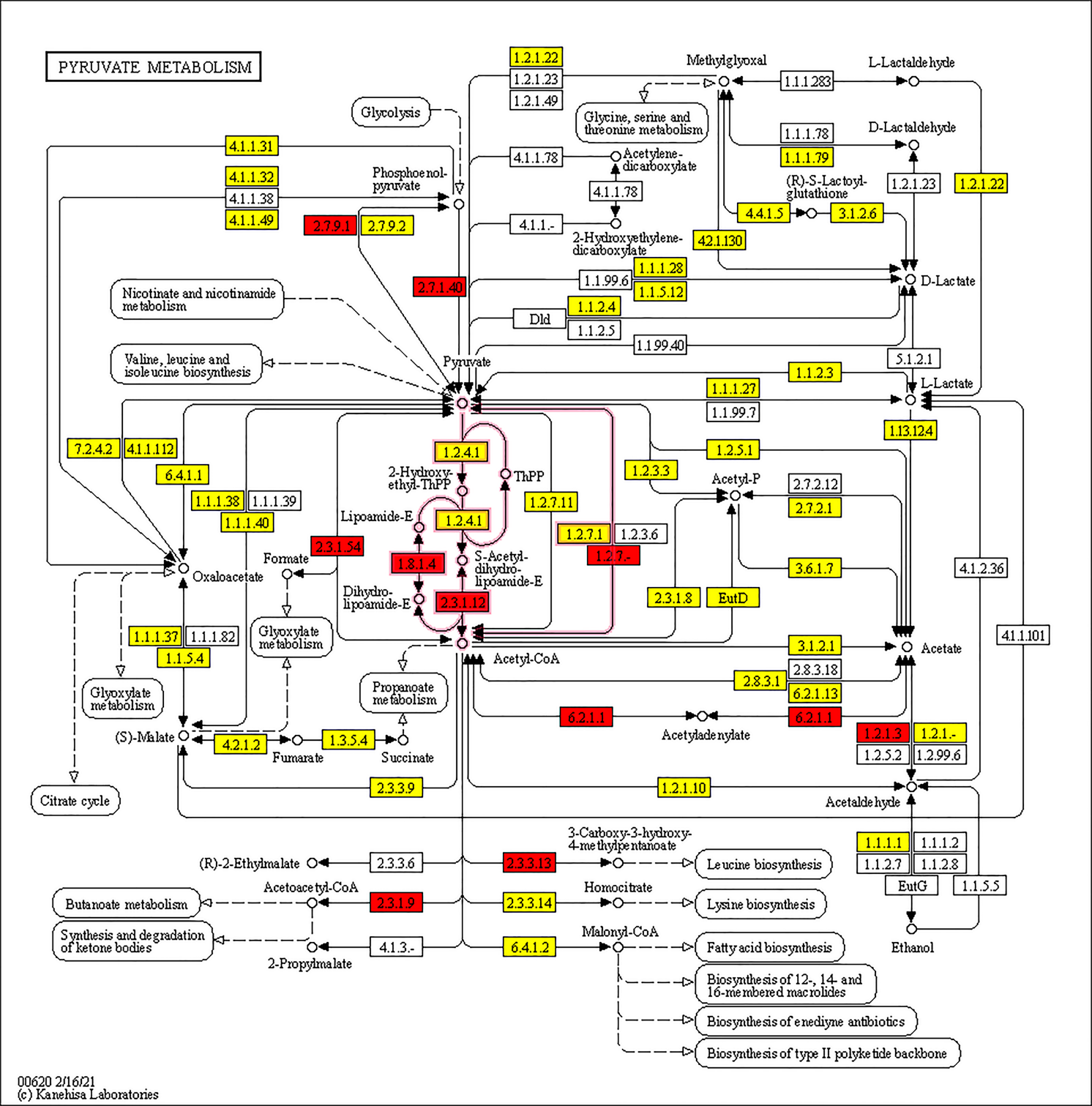


Boxes with color represent the annotated enzymes of biliary microbiota in patients with choledocholithiasis. The top ten enzymes in terms of abundance were indicated in red, while others were in yellow.

**Supplementary** **Figure S2**. Enriched pathways in glycolysis/gluconeogenesis of biliary microbiota in patients with choledocholithiasis


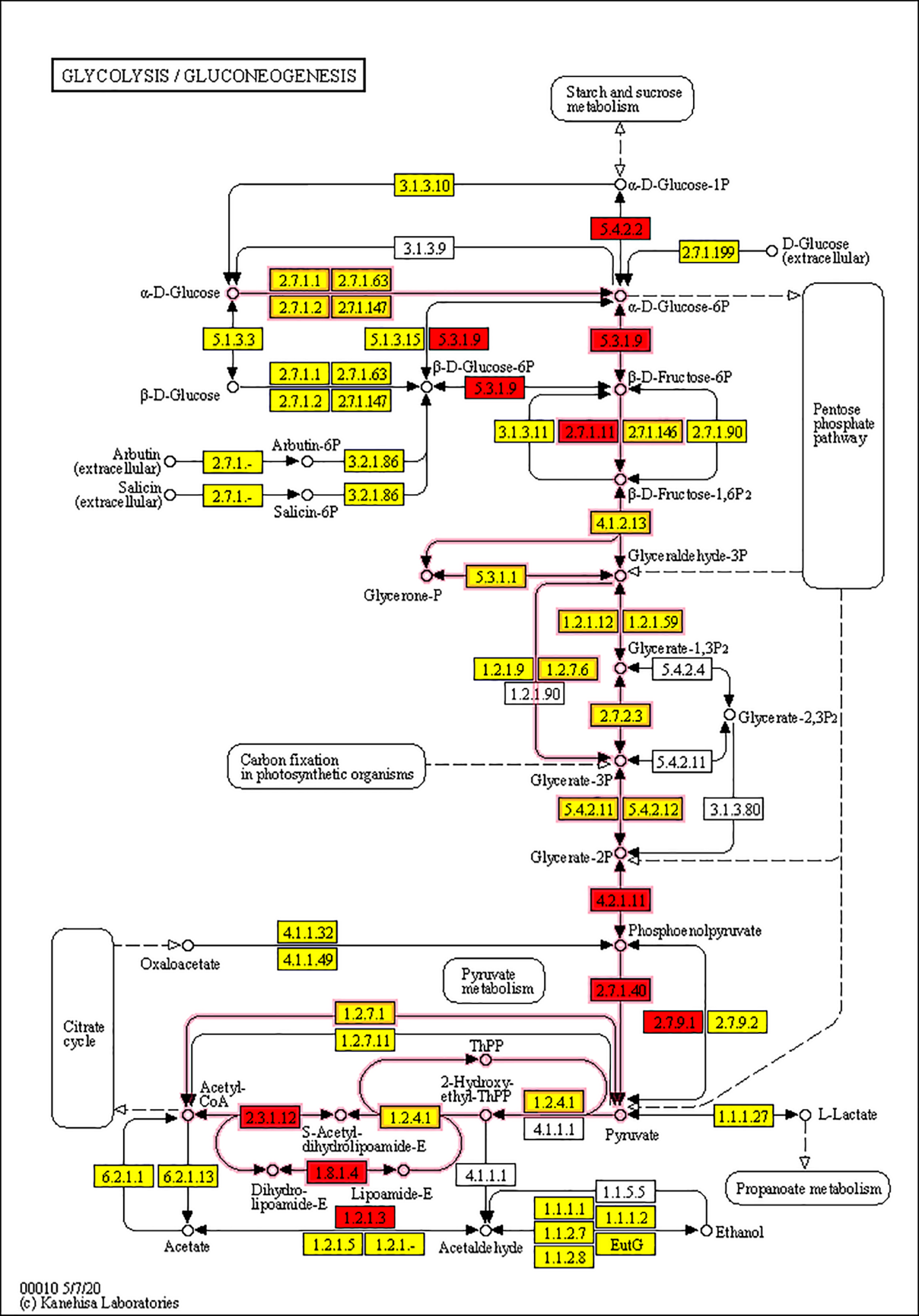


Boxes with color represent the annotated enzymes of biliary microbiota in patients with choledocholithiasis. The top ten enzymes in terms of abundance were indicated in red, while others were in yellow.

**Supplementary** **Figure S3**. Enriched pathways in amino sugar and nucleotide sugar metabolism of biliary microbiota in patients with choledocholithiasis


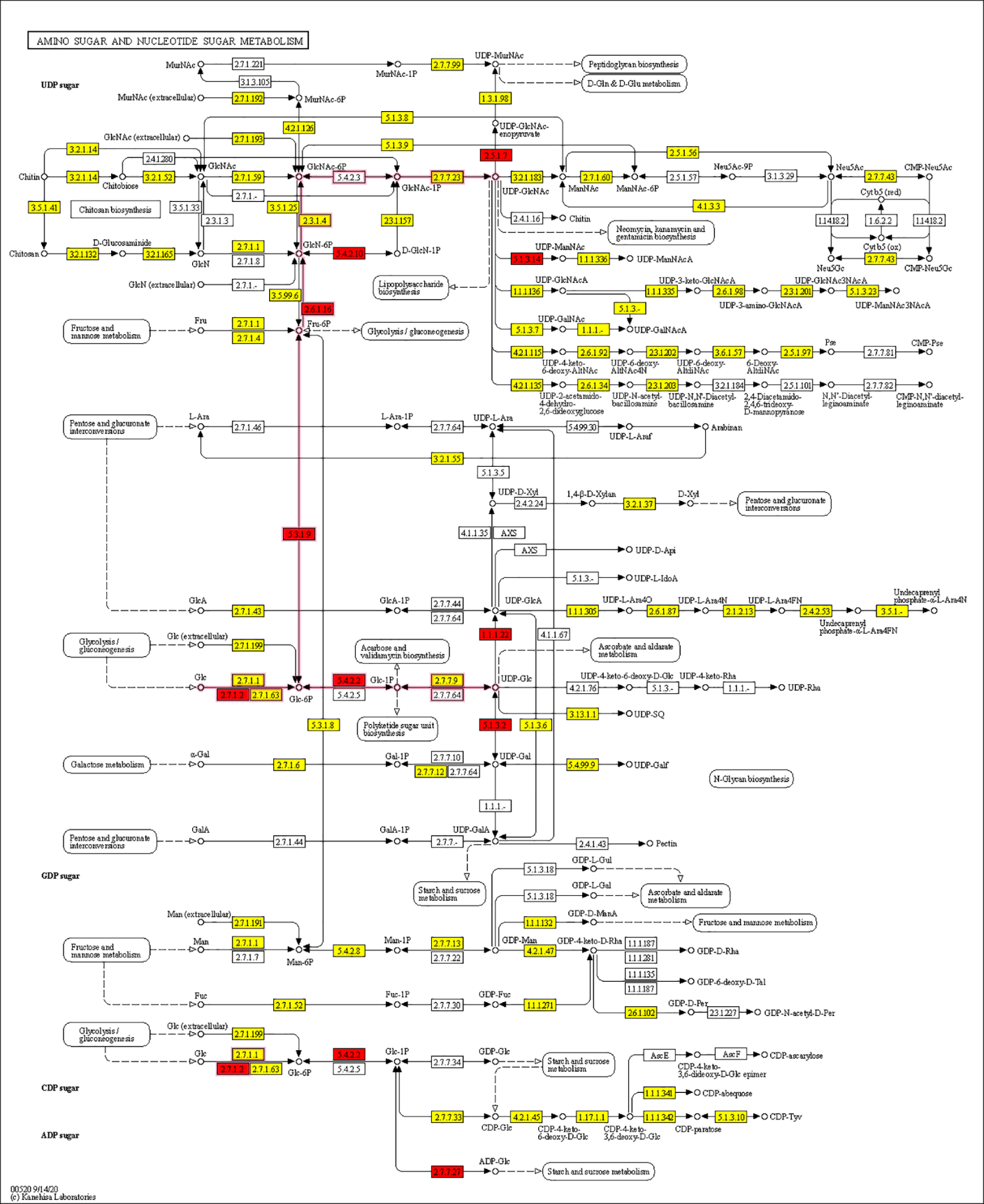


Boxes with color represent the annotated enzymes of biliary microbiota in patients with choledocholithiasis. The top ten enzymes in terms of abundance were indicated in red, while others were in yellow.

**Supplementary** **Figure S4**. Enriched pathways in alanine, aspartate and glutamate metabolism of biliary microbiota in patients with choledocholithiasis


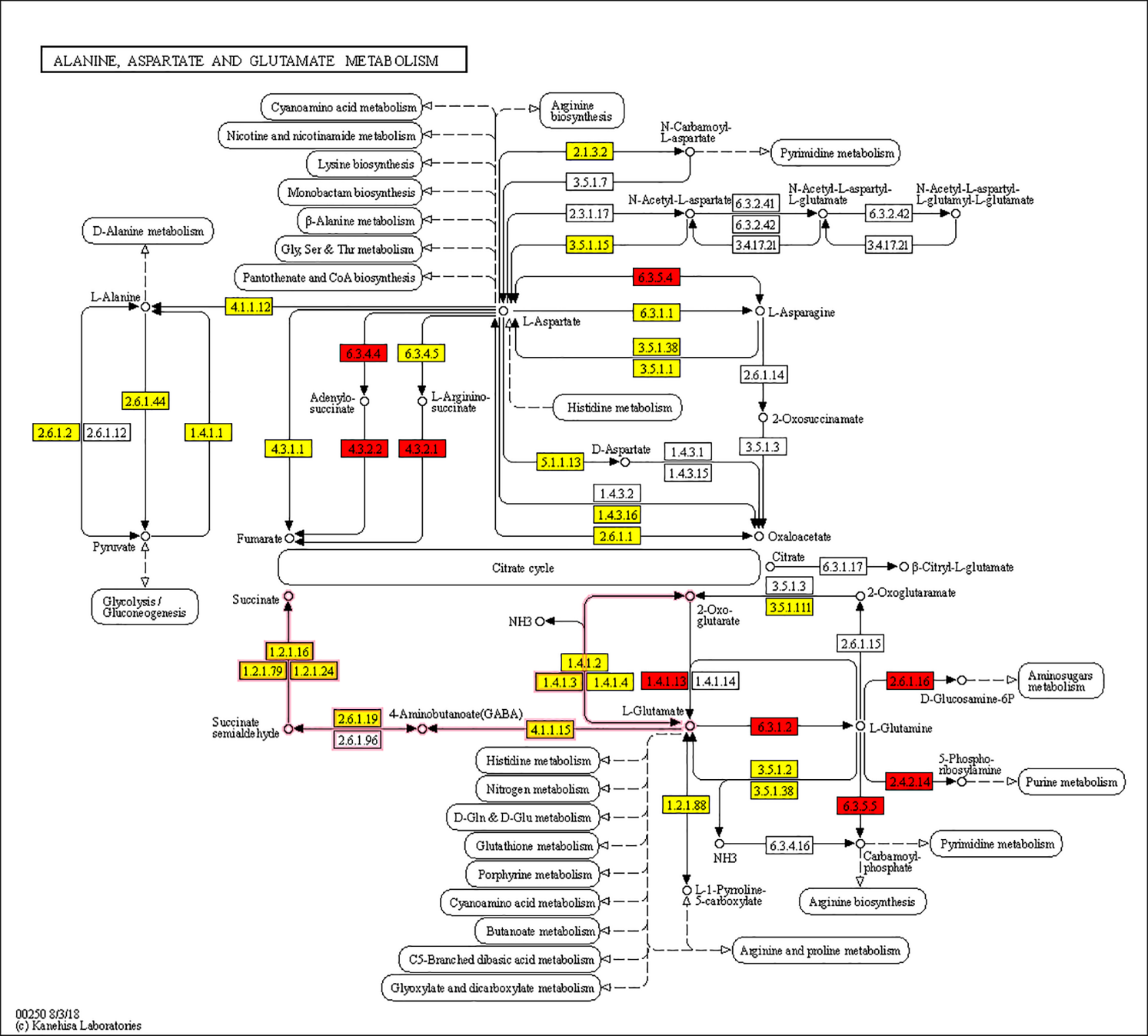


Boxes with color represent the annotated enzymes of biliary microbiota in patients with choledocholithiasis. The top ten enzymes in terms of abundance were indicated in red, while others were in yellow.
